# The closer the better: does better access to outpatient care prevent hospitalization?

**DOI:** 10.1007/s10198-019-01043-4

**Published:** 2019-03-15

**Authors:** Péter Elek, Tamás Molnár, Balázs Váradi

**Affiliations:** 1 Department of Economics, Eötvös Loránd University (ELTE), Pázmány Péter sétány 1/A, Budapest, 1117 Hungary; 2 Institute of Economics, “Lendület” Health and Population Research Group, Centre for Economic and Regional Studies, Hungarian Academy of Sciences, Budapest, Hungary; 3 Budapest Institute for Policy Analysis, Budapest, Hungary

**Keywords:** Administrative panel data, Inpatient care, Outpatient care, Potentially avoidable hospitalization, Quasi-experiment, Substitution, C23, C26, I10

## Abstract

In 2010–2012, new outpatient service locations were established in poor Hungarian micro-regions. We exploit this quasi-experiment to estimate the extent of substitution between outpatient and inpatient care. Fixed-effects Poisson models on individual-level panel data for years 2008–2015 show that the number of outpatient visits increased by 19% and the number of inpatient stays decreased by 1.6% as a result, driven by a marked reduction of potentially avoidable hospitalization (PAH) (5%). In our dynamic specification, PAH effects occur in the year after the treatment, whereas non-PAH only decreases with a multi-year lag. The instrumental variable estimates suggest that a one euro increase in outpatient care expenditures produces a 0.6 euro decrease in inpatient care expenditures. Our results (1) strengthen the claim that bringing outpatient care closer to a previously underserved population yields considerable health benefits, and (2) suggest that there is a strong substitution element between outpatient and inpatient care.

## Introduction

How to best allocate limited public resources across outpatient and inpatient healthcare services to achieve maximum improvement in health outcomes is one of the perennial questions of health policy all over the world.

To inch closer to answering that question, we have to understand, disentangle, and accurately measure the relationships between those two levels of care. Does the provision of more outpatient care, while itself improving health outcomes, also generate more hospitalization episodes and/or make them longer, or, to the contrary, does it help to avoid costlier inpatient care later on? What are the respective and aggregate changes in health care expenditures? In this paper, we use panel data from a quasi-experimental setting provided by an expansion of specialist outpatient care in Hungary between 2010 and 2012, greatly improving access, to contribute to answering those questions. Besides observational or cross-sectional studies, the earlier quasi-experimental literature mainly uses data from the United States, and hence, little is known about the substitution/complementation effects in countries whose health care sector is characterised by different institutional and regulatory frameworks and financing arrangements.

At the highest level of abstraction, nationwide health policy planning is about maximizing health outcomes of the population constrained by limited public and private resources. This is done through financing many functional channels of the health care system, but, in OECD countries, most expenditure goes to curative and rehabilitative care, and, within that, two of the most important functions are outpatient care, upon which 1.2–7.5% of gross domestic product (GDP) is expended; and inpatient care, with 1.5–3.4% of GDP (2015 data from [17]). Given these enormous expenses, the importance of any reliable evidence that can contribute to even a marginal improvement of health outcomes by a better allocation of resources across these two subsectors cannot be overstated. Such evidence can help policy makers to decide whether additional public resources are put to better use by being channelled toward expanding outpatient or inpatient care. In what follows, we first present the possible mechanisms of substitution and complementation and the empirical literature so far, then the Hungarian context, followed by the data, the methods, our results and, finally, our conclusions.

## Mechanisms of substitution and complementation

What are the possible theoretical mechanisms of interaction between inpatient and outpatient care? Fortney et al. [8], building on the work of Starfield [21] and others, identify the following mechanisms of substitution (i.e. more outpatient care decreases hospitalization) and complementation (more outpatient care reduces more inpatient care).

Mechanisms of substitution:

- Early detection of an illness in outpatient care can make treatment possible at that level and obviate the need for hospitalization. This substitution mechanism, they claim, could have both short-term (e.g. prevention of hospitalization for asthma by prevention and early treatment of exacerbations) and long-term effects (e.g. prevention of stroke by the treatment of hypertension).
- The management of chronic health conditions in outpatient care (e.g. routine testing or patient education) can also prevent or at least delay the need for inpatient care—control of blood sugar to avert kidney failure in patients with diabetes mellitus is a classic example of this.
- Depending on the rules and incentives built into the health care system of the country in question, doctors in outpatient care could have a formal gate-keeping role, as well: in many cases, their referral can be required for hospitalization.

Mechanisms of complementation:

- Treatment in outpatient care might call for supplemental or ancillary care provided in hospitals (e.g. diagnostic laboratory tests).
- The detection in outpatient care of illnesses (e.g. cancer, serious mental illness) that are best treated by a specialist, in hospital. This mechanism could especially affect patients who have not used primary care services for a long period of time and who have a greater number of undetected illnesses.
- The identification (through close monitoring) of acute episodes of chronic illnesses that require specialty or inpatient treatment. This mechanism is particularly relevant for disorders with symptoms that may fluctuate in severity over time (e.g. angina or major depressive disorder).

The empirical literature is rather mixed in terms of whether the substitution or the complementation effect dominates. Miller [15] analysing a Massachusetts reform (a health insurance reform was introduced that differentially affected the costs of outpatient and inpatient care) and Rubinstein et al. [20] analysing the effects of a reorganization to increase access to primary care for veterans in Virginia both found a drop in hospitalization in response to more access to primary care. Other papers also found substitution effects in cross-sectional settings [1, 6, 10, 19].

On the other hand, Kaestner and Sasso [11] found that, in the US, an increased outpatient spending was associated with more hospital admissions; the Rand and the Oregon health insurance experiments also showed that improving the availability of medical services through a more generous health insurance coverage was associated with an increase in the use of emergency room services and hospitalization [7, 16].

A third group of studies found neither substitution nor complementation effects. Looking into the same Massachusetts reform as Miller [15], Kolstad and Kowalski [12] found that gaining insurance was associated with a decrease in hospital admissions through emergency department, an increase in hospital admissions through other channels, and no change in total hospitalizations. The instrumental variables analysis by Fortney et al. [8] indicated that an increase in primary care encounters was associated with a decrease in specialty medical encounters, but was not associated with an increase in physical health admissions or outpatient costs.

One promising method to try to sharpen the results in the empirical literature, exemplified by Duscheiko et al. [3], has been to narrow down the focus upon hospitalizations for conditions considered especially sensitive to timely and effective management in primary care; e.g. Kolstad and Kowalski [12], whose inconclusive results put them in the “no effect” camp above, actually find a substitution effect when zooming in on the effects upon preventable hospitalization.

## Institutional context

In addition to being, in sum, rather inconclusive, many of these studies are also observational or cross-sectional, making the establishment of causal relationships hard. In the case of papers based on a quasi-experimental or experimental setup, the source of variation that makes identification possible consists in changes in the financing (insurance) mechanism alone and almost all of them examine the US. Our source of variation is different and our evidence comes from a very different, but, by no means, internationally unique institutional setting, shared by most post-communist EU member states (e.g. Poland and the Czech Republic) and the countries emerging from the Soviet Union like Russia and Ukraine [14]. In such countries, our research question has never been addressed before.

Hungary is a post-communist EU member state of slightly less than 10 million inhabitants with a single-payer health insurance and de facto universal coverage [5]. In 2015, Hungary spent 7.2% of its GDP on health care, 1.8% of the GDP on outpatient (including government- and household-financed primary and specialist outpatient) and 1.9% of the GDP on inpatient care [17]. The basic benefit package is free of out-of-pocket payments for the patients at the point of care (including outpatient care), although informal gratuity payments are widespread. Primary care by general practitioners is financed by capitation; most outpatient services are financed by the budget based on fee-for-service points, under a system that scores procedures on the basis of their complexity and resource requirements, whereas inpatient services, almost exclusively provided in state-run and -financed hospitals, are reimbursed through a combined payment system based on diagnosis-related groups (acute care) and per diem rates (chronic care).

The relatively high share of outpatient care in provision and financing is due to the heritage of the Semashko-type healthcare system, common in countries once under Soviet dominance. Central to that model was a multi-tiered system of care with a strict referral system and strongly differentiated network of service providers, with outpatient specialist care, provided in dedicated polyclinics and thus separated from primary care, one of the distinct tiers of healthcare provision [9, 13]. Concentrating on the relationship between this type of care and inpatient care can, arguably, provide more precise information on substitution/complementation than what can be obtained in healthcare systems where data on primary and specialised outpatient care are lumped together. Given the institutional arrangement in Hungary, and to avoid confusion, in what follows, we will refer to “specialist outpatient” care, or, for brevity, “outpatient care” as disjoint from “primary care”, whereas we retain the term “ambulatory care” when referring to the general international literature that uses this term subsuming primary care, as well. We will also address another subset of outpatient care, “1-day ambulatory care” later on.

The health status of the Hungarian population is among the poorest in the EU with a life expectancy at birth of 75.7 years, tailing the EU average by 4.9 years, with even worse parameters in rural micro-regions in which the intervention which we use for identification took place.

The intervention which we base our quasi-experimental specification on is the same as used in Elek et al. [5]. Between 2010 and 2012, around 430,000 people gained better access to specialist outpatient care in Hungary when the government created outpatient units in 20 rural micro-regions, which previously lacked capacity. The investments were funded by the Social Infrastructure Operative Programme (SIOP) 2.1.2. of the European Union. Locations for the new units were selected based on the applications of municipalities, making a case for need and demand.^1^ Funding accounted for 500–1000 million HUF (2–4 million euros) per unit, generally covering 90–95% of the costs of the establishment of the new units to the municipalities if they complied with a set of administrative requirements (e.g. providing a minimum of services for a minimum of hours/month, keeping the unit in operation for at least 5 years). Competition for scarce funds was not an issue: sufficient funds were allocated to be able to subsidize all likely applicants eligible under those rules. The newly created units (all still in operation as of 2016) provide comprehensive service for the population of the micro-regions with at least 14 separate specialties at each location. As a result, basic specialist outpatient care in the following four specialties: internal medicine, surgery, obstetrics—gynaecology, and pediatrics may now be reached by around 310,000 more people by car in 20 min than before.

At the same time, the other parts of Hungary experienced relatively few changes in the management of outpatient care between 2008 and 2015. Hence, an appropriate control group of micro-regions could be identified, in which the health care indicators may be compared to those in the micro-regions where new outpatient service locations were established (the “treated” micro-regions). The impact of the improvement in accessibility can then be estimated as the difference between the changes in the treated and control groups, with a difference-in-difference-type analysis.

It is the treatment that we use in the paper to identify the sign, the magnitude, and the lag of the effect of more outpatient treatment upon hospitalization at the individual level.

## Data and descriptive statistics

We use anonymized individual-level administrative data on inpatient stays and specialist outpatient visits, exclusively provided to us for this research project by the Hungarian National Healthcare Services Centre (ÁEEK). Data cover years 2008–2015 for the population of 20 treated and 20 control micro-regions with approximately 1,060,000 people in Hungary (around 10% of the population of the country). The control micro-regions were chosen with propensity score matching to approximate the pre-treatment demographic, socio-economic, and health characteristics of the treated micro-regions. Elek et al. [5] provide the details on the matching procedure as well as on the treated control balance in terms of the observed pre-treatment characteristics.^2^ The balance is satisfactory in most variables, although there remains a slight—statistically not significant—difference in pre-treatment outpatient care provision. The number of weekly specialist outpatient consultation hours per 1000 residents averaged to 0.6 in the treated and 1.2 in the control micro-regions in 2008, but the latter was still very small compared to the average value of all non-treated micro-regions in Hungary (3.8). We will control for this pre-treatment difference by the fixed-effects models.

The annual panel data set used in our analysis contains for each person-year the number of inpatient stays (and of its certain subgroups, see below), the number of specialist outpatient visits (and of its certain subgroups), the estimated inpatient and outpatient care expenditures, as well as demographic information such as gender, year of birth, and settlement of residence.^3^ Year of death is also recorded for those who died during the period. We omit newborns from the sample, and, hence, restrict the analysis to those at least 2 years of age.

Annually, around 13% of the population of the control micro-regions was hospitalized. We also define potentially avoidable hospitalization (PAH), i.e. hospitalization due to ambulatory care sensitive conditions (ACSCs), based on the ICD-10 category of the primary diagnosis of the inpatient episode. Our main definition for PAH follows Purdy et al. [18] as described in detail by Eggli et al. [4].^4^ According to this definition, around 2.4% of the population was hospitalized due to an ACSC in a given year. We classify this category into the following subgroups (see "Appendix 1" for details):

- cardiology-related conditions (angina, congestive heart failure, and hypertension) (0.8%),
- pulmonology-related conditions (asthma and COPD) (0.6%),
- diabetes complications (0.3%),
- conditions due to non-adequate specialist outpatient care (e.g. ear, nose, and throat infection) (0.3%), and
- conditions due to non-adequate primary care (e.g. influenza) (0.6%).

**Fig. 1:**
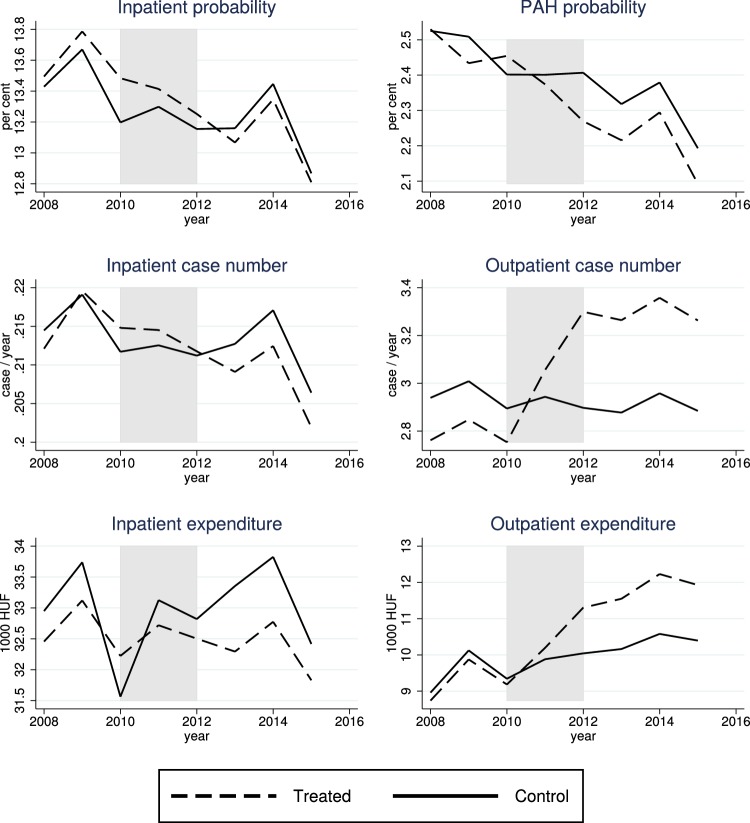
Per capita use of inpatient and (non-laboratory) outpatient care in the treated and control micro-regions

Figure 1 shows that hospitalization case number, hospitalization probability, as well as PAH probability decreased more in the population of the treated group than of the control group after 2010–2012, when the new outpatient units started to operate in the treated micro-regions.^5^ Most new units were established in 2011. The difference between the treated and control values was slightly positive or roughly zero before 2011, but became negative afterwards. We will also examine certain other subgroups of hospitalization such as acute and chronic episodes.

Meanwhile, according to Fig. 1, the number of outpatient visits jumped high in the treated compared to the control group after 2010–2012. The levels and trends are consistent with outpatient capacities: due to some existing outpatient units in the control micro-regions, outpatient care use was slightly higher in the control than in the treated group before 2011, but this difference quickly reversed when the new outpatient units emerged in the treated micro-regions. We also note that, before 2011, outpatient case numbers in the treated group were well below the national average and also below (by 20–25%) those rural micro-regions that already had substantial outpatient capacities [5], so the sudden increase was just a catch-up from a low initial level in an underserved population.

We will specifically examine outpatient visits associated with certain ACSCs such as those in cardiology, pulmonology, or diabetes, defined by the ICD-10 code of the outpatient event. We hypothesize that a growing ratio of patients treated in outpatient care with such conditions may have caused the decreased prevalence of PAH.

Finally, the lower two graphs in Fig. 1 show that, while outpatient expenditures increased, the estimated inpatient expenditures decreased in the treated compared to the control micro-regions.^6^

Obviously, outpatient care use and inpatient care use are strongly correlated on the individual level. In the control micro-regions, patients hospitalized in a given year visited (non-laboratory) outpatient care 2.9 times more often in the previous year than non-hospitalized patients, and the difference persists when age and gender are controlled for. However, these cross-sectional correlations are non-causal. Estimation of a causal relationship between outpatient and inpatient care requires a quasi-experiment such as the establishment of the new outpatient locations in our case. Therefore we apply a difference-in-difference-type analysis to measure the treatment effect (and check the pre-treatment parallel trends in the treated and control group with a placebo test). In support of our identification approach, it is important to also add that, although hospital capacities had been curtailed to save costs in 2007, before the time period under scrutiny, there was virtually no policy-driven change in the supply of inpatient care during the time-span which we analyse. Still, we use three explanatory variables to control for possible exogenous changes in health care supply in the examined period: the number of wider regional (county-) level number of hospital beds; the ratio of unfilled GP practices in the settlement of the individual; and the availability of special 1-day ambulatory services (aimed at providing certain treatments in internal care, neurology, and physiotherapy) that started to operate in some treated and control micro-regions in the examined period (see "Appendix 3" for details, and Table 10 in "Appendix 4" for descriptive statistics of these variables in the treated and control groups).

Beyond an impact assessment of the establishment of the new outpatient units, we use this quasi-experiment to estimate the structural effect of bringing outpatient care 1 min closer to the residence of the individual on hospitalization. Therefore, we define the travel time (in minutes) needed to reach the nearest outpatient unit by car from the settlement of each individual. This distance measure decreased in the treated micro-regions from 24 min in 2008 to 10 min in 2012, while it was essentially unchanged (21 min on average) in the control micro-regions.

## Methods

### Effects of the new outpatient locations

For person *i* in year *t*,  let $y_{it}$ denote the number of hospital admissions (or the number of its various subcategories), $d_{it}$ the dummy variable that equals one for the population of the treated micro-regions after the establishment of the new units and zero otherwise (i.e. zero always for the control group and before the establishments for the treated group), $T_{it}$ the calendar year dummies, and $z_{it}$ the control variables (a cubic function of individual age and the health care supply variables described above). In our baseline models, we estimate the effect of the treatment on the expected number of admissions, $E (y_{it} ),$ with fixed-effects (FE) Poisson, and, for robustness check, with FE linear models. We also estimate the probability of hospitalization, $\Pr (y_{it}>0),$ with FE logit:

1 $$\begin{aligned} E (y_{it} )= \exp (\beta _d^{\text {poi}} d_{it}+ \beta _{T}^{\text {poi}} T_{it} + \beta _z^{\text {poi}} z_{it} + c^{\text {poi}}_{i}) \end{aligned}$$

2 $$\begin{aligned} E (y_{it} )= \beta _d^{\text {lin}} d_{it}+ \beta _{T}^{\text {lin}} T_{it} + \beta _z^{\text {lin}} z_{it} + c^{\text {lin}}_{i} \end{aligned}$$

3 $$\begin{aligned} \Pr (y_{it}>0)= {\text {logit}} (\gamma _d d_{it}+ \gamma _{T} T_{it} + \gamma _z z_{it} + c^{\text {logit}}_{i} ), \end{aligned}$$

where $\beta$-s and $\gamma$-s are the parameters, $c_i$-s denote the individual-level heterogeneity, and ${\text {logit}}$ is the logistic function. We treat $c_i$-s, the fixed effects, as completely unrestricted. They control for, among others, any pre-treatment differences in the health status of the individuals, and also for any time-constant differences in individuals such as their gender. For the estimation of FE Poisson, FE linear, and FE logit models see Wooldridge [23].

We estimate further models with different dependent variables:

- Models (1)–(3) on the number of outpatient cases (FE Poisson and FE linear) and on the probability of receiving outpatient care (FE logit), for person *i* in year *t*, and on the ACSC-related outpatient subcategories.
- FE linear models on inpatient and outpatient expenditures of person *i* in year *t*.

Moreover, we investigate the heterogeneity of the effect of new outpatient locations on inpatient stays with various treatment interaction models (in FE Poisson and FE logit specifications):

- first, the treatment dummy is interacted with gender and age groups to examine potential heterogeneity across these categories;
- second, the treatment dummy is interacted with the indicators of local supply of inpatient care such as the travel time between the micro-region and the nearest (substantial) hospital^7^ or the capacity utilization rate of the beds in the nearest hospital;
- third, the (changing) travel time to the nearest outpatient service location is used as an additional explanatory variable beyond the treatment dummy to examine the effects of the heterogenous improvement in outpatient availability across settlements.

FE models estimate the treatment effect using within-person variation, i.e. by calculating how a person’s probability and frequency of health care use changed as a result of the treatment compared to the control group. These models usually give more credible inference than e.g. pooled methods on panel data, because it is difficult to control for all individual-level pre-treatment differences in the latter models. However, if there is a slight change in the probability of death in the treated compared to the control group (large effects are unlikely to occur in the 3–4 years after the establishments, which we test with a pooled logit model), FE and pooled models may yield different estimates, because dying patients are selected out of the sample at a slightly different rate in the two groups. Therefore, we perform two robustness checks. First, we estimate pooled Poisson and logit models on inpatient and outpatient care use. For instance, the pooled Poisson specification is defined as follows:

4 $$\begin{aligned} E (y_{it}) = \exp (\beta _d^{\text {poi}} d_{it}+ \beta _{T}^{\text {poi}} T_{it} + \beta _z^{\text {poi}} z_{it} + \beta _w^{\text {poi}} w_{it}), \end{aligned}$$

where $w_{it}$ now contains additional controls such as gender (interacted with age) and the micro-region of the individual. Second, we estimate the FE Poisson and logit models (1)–(3) on the subsample of those who did not die during the 8 years long period.

Besides, we estimate dynamic treatment effects with versions of the above models. Let $l_{it}^{(k)}=d_{i,t-k}-d_{i,t-k-1}$ indicate the period exactly *k* years after the establishment of the new outpatient location in the micro-region of person *i*. Then, in the FE Poisson equation:

5 $$\begin{aligned} E (y_{it} )= & {} \exp (\beta _0 l_{it}^{(0)}+ \beta _{1} l_{it}^{(1)} + \beta _{2} l_{it}^{(2)}\nonumber \\&+ \ \beta _{3+} d_{i,t-3} + \beta _{T} T_{it} + \beta _z z_{it} + c_{i} ), \end{aligned}$$

$$\beta _{k}$$$$(k=0, 1, 2)$$ measure the treatment effect exactly after *k* years, and $$\beta _{3+}$$ shows the effect after 3 or more years. More lags cannot be included, because only about 4 years have passed after the initiation of the new outpatient locations. We use hospitalization, PAH, non-PAH case numbers, and probabilities as well as outpatient case numbers as dependent variables in the dynamic models.

The parallel line assumption is crucial behind these models, i.e. that, after netting out the effect of the control variables, the outcome variables in the treated micro-regions would have changed in the absence of the treatment in the same way as they actually did in the control micro-regions. Therefore, we estimate a version of (5) for years 2008–2010, before the treatment:

6 $$\begin{aligned} E (y_{it} )= & {} \exp (\beta _{g,2008}\cdot g_{i}\cdot I_{\{t=2008\}}+ \beta _{g,2009}\cdot g_{i}\cdot I_{\{t=2009\}} \nonumber \\&+ \ \beta _{T} T_{it} + \beta _z z_{it} + c_{i} ), \end{aligned}$$

where $g_i$ denotes the group of the (later) treated micro-regions, $I_{\{t=k\}}$ the calendar year dummies, and we test whether $\beta _{g,2008}=\beta _{g,2009}=0,$ i.e. the group differences—after controlling for the explanatory variables—are the same in 2008, 2009, and 2010, where the latter difference is captured by the individual fixed effects.

### Substitution between outpatient and inpatient care

The most important advantage of the establishment of the new outpatient units is that we can exploit this quasi-experiment to estimate the causal impact of more frequent outpatient care use on inpatient care use. Formally, we estimate fixed-effects linear instrumental variable (FE IV) models of the form:

7 $$\begin{aligned} E (y_{it}) = \delta _x x_{it}+ \delta _{T} T_{it} + \delta _z z_{it} + c_{i}, \end{aligned}$$

where, in the baseline IV specification, $y_{it}$ is the number of hospital admissions and $x_{it}$ is the number of outpatient care visits for person *i* in year *t*,  and $x_{it}$ is instrumented with $d_{it},$ the treatment dummy. The reduced form and the first stage of this model are the FE linear models (2) of inpatient and outpatient case numbers, respectively. Here, we use a linear model, because fixed-effects and instrumental variables are computationally not straightforward to incorporate simultaneously in a Poisson specification.

We also estimate the substitution/complementation effect in terms of outpatient and inpatient expenditures, i.e. using inpatient expenditures as the dependent variable and outpatient expenditures as the endogenous explanatory variable, instrumented by the treatment variable, in an FE IV model.

Furthermore, the long panel data set at our disposal enables us to measure the dynamics using contemporary and lagged outpatient care use variables as endogenous explanatory variables, instrumented by the contemporary and lagged treatment dummies. Formally, we estimate FE IV models of the form:

8 $$\begin{aligned} E(y_{it}) = \delta _0 x_{it}+ \delta _1 x_{i,t-1} + \delta _{T} T_{it} + \delta _z z_{it} + c_{i}, \end{aligned}$$

where $x_{it}$ and $x_{i,t-1}$ are (jointly) instrumented by $d_{it}$ and $d_{i,t-1}.$ Here, we only include one lag, since the pre-treatment period contains only 2 or 3 years for most micro-regions.

## Results

Table 1 presents the estimated treatment effects on the use of inpatient and outpatient care. Descriptive statistics of the variables used in the models are shown in Tables 8, 9 and 10 of "Appendix 4". The left panel displays the annual baseline probabilities of receiving a certain type of care in the control group, along with the effects of the treatment on these probabilities. Odds ratios (i.e. $\exp (\gamma _d )$) are shown, which roughly correspond in the case of inpatient care to multiplicative changes in probabilities, because hospitalization is relatively rare in the population. The right panel gives the baseline case numbers (per 100 inhabitants), the multiplicative effects of the treatment according to the FE Poisson models, i.e. $\exp (\beta _d^{\text {poi}} ),$ and the additive effects $\beta _d^{\text {lin}}$ according to the FE linear models. The lower panel of the Table contains the baseline health expenditure values in the control group and how the treatment affects them.

**Table 1:** Effects of the establishment of new outpatient locations

	Probabilities			Case numbers				
	Baseline (%)	FE logit		Baseline (/100)	FE Poisson		FE linear	
		Odds ratio			Multipl. effect		Effect (per 100)	
		Est.	SE		Est.	SE	Est.	SE
Inpatient care								
Overall	13.3	0.985**	(0.006)	21.3	0.984**	(0.006)	− 0.63***	(0.12)
Not PAH	11.9	0.998	(0.006)	18.4	0.991	(0.007)	− 0.38***	(0.12)
PAH	2.4	0.932***	(0.012)	2.9	0.950***	(0.013)	− 0.25***	(0.033)
Cardiology	0.80	0.906***	(0.019)	0.93	0.909***	(0.020)	− 0.095***	(0.018)
Pulmonology	0.58	1.005	(0.027)	0.74	1.035	(0.030)	− 0.066***	(0.017)
Diabetes	0.27	0.934**	(0.032)	0.31	0.945	(0.033)	− 0.017*	(0.011)
Spec. care spec.	0.31	0.932**	(0.030)	0.32	0.935*	(0.033)	− 0.021**	(0.010)
Prim. care spec.	0.59	0.981	(0.024)	0.61	0.975	(0.027)	− 0.047***	(0.014)
Outpatient care								
Overall non-lab.	54.6	1.232***	(0.005)	293.0	1.185***	(0.004)	53.0***	(0.94)
Cardiology	5.8	1.290***	(0.011)	11.0	1.209***	(0.011)	2.8***	(0.11)
Pulmonology	4.2	1.203***	(0.013)	8.7	1.043***	(0.012)	0.25**	(0.097)
Diabetes	2.3	1.325***	(0.025)	5.3	1.204***	(0.015)	1.2***	(0.073)
Overall lab.	31.4	1.107***	(0.005)	105.0	1.148***	(0.006)	17.0***	(0.55)
Cardiology	1.7	2.586***	(0.037)	2.9	1.786***	(0.035)	2.8***	(0.065)
Pulmonology	0.33	1.349***	(0.050)	0.70	1.126**	(0.060)	− 0.16***	(0.030)
Diabetes	0.76	3.118***	(0.079)	1.4	2.038***	(0.050)	1.2***	(0.036)

				Expenditures				
				Baseline (1000 HUF)			FE linear	
							Effect	SE
Inpatient				32.6			− 0.82***	(0.26)
Outpatient				9.9			1.28***	(0.040)

Probability of not PAH refers to the probability that all inpatient stays are not PAH in a year

Baseline: the average values in the control group

Spec./prim. care spec.: PAH due to non-adequate specialist outpatient/primary care

Cluster-robust standard errors (SE) are displayed for all models apart from FE logit

Controls: fixed effects, cubic age, calendar year dummies, health care supply variables

Number of observations: 7,412,000. Number of periods: 8. Number of people: 1,037,000

*PAH* potentially avoidable hospitalization

***$$p<0.01,$$ **$$p<0.05,$$ *$$p<0.1$$

### Inpatient care

The upper panel of Table 1 shows that both the odds of hospitalization and the number of hospital admissions decreased by about 1.5% as a result of the establishment of new outpatient units. In relative terms, the FE linear model gives an even stronger negative effect at the baseline value (− 0.63 per 21.3, i.e. − 2.9%). Non-ACSC related hospitalization remained essentially unchanged (although the FE linear model shows a small absolute reduction), while ACSC-related inpatient stay decreased substantially (odds by 7%, case number by 5–8%). This was driven by a reduction in cardiology, diabetes-related, and specialist care specific PAH, which have ORs around 0.91–0.93, but the FE linear specification gives negative coefficients for all the categories.

According to the lower panel of Table 1, per capita inpatient expenditures decreased by 820 HUF (2.8 euros) or by 2.5% of the average expenditure, at about the rate of the reduction of inpatient case numbers. This suggests a roughly constant case mix (expenditure per inpatient episode).

### Outpatient care

According to the middle panel of Table 1, the improved accessibility of ambulatory care increased outpatient case numbers by 19% in the non-laboratory and 15% in the laboratory segment.^8^ Cardiology and diabetes-related outpatient case numbers grew faster, while pulmonology-related case numbers increased slower than average. Remarkably, the number of laboratory tests with ACSC-related cardiology and diabetes diagnoses roughly doubled, and the ratio of patients having annually at least one laboratory test with such diagnoses approximately tripled. Since the standard protocol for the treatment of diabetes mellitus includes regular blood tests such as HbA1c screening to check long-term blood glucose levels, this suggests that a growing number of diabetes patients became treated according to the protocol, implying a health gain for the population.

Finally, the lower panel of the table shows that per capita outpatient expenditures increased by about 1300 HUF (4.4 euros), which is larger in absolute terms than the decrease of inpatient care expenditures.^9^ The additional costs make up about 13% of the average expenditure, in good accordance with the estimated effect on outpatient case numbers.

### Heterogeneity and robustness checks

According to the heterogeneity analyses (not shown here in detail), the relative treatment effect—the logit odds ratio or the Poisson multiplicative effect—is not significantly different across gender and age groups. Similarly, the local supply of inpatient care—the distance to the nearest hospital or the capacity utilization rate of beds there—does not significantly influence the effect of the new outpatient locations on inpatient stays (*p* values of the treatment interaction terms exceed 0.1 in all the specifications). At the same time, the differential reduction of the distance to the nearest outpatient location—which varies across the treated micro-regions—has an additional explanatory power beyond the treatment dummy on the reduction of inpatient stays. In other words, the number of inpatient stays decreased statistically significantly more in settlements with greater improvement in travel time. These results—effect heterogeneity in the outpatient and homogeneity in the inpatient dimension—suggest the exclusive role of the outpatient channel in the reduction of inpatient stays, and, hence, give indirect evidence for the exogeneity of the treatment dummy as an instrument in the substitution analysis between outpatient and inpatient care below.

Table 2 displays the effects of the reduction in travel time to the nearest outpatient care provider by car on hospitalization.^10^ A 10-min reduction in travel time decreases PAH much more strongly than non-PAH ($\hbox {OR}=0.965$ vs. 0.989, i.e. the change in odds is threefold for PAH), and cardiology and diabetes-related PAH are particularly influenced.

**Table 2:** Effects of bringing outpatient care closer by 10 min with car

	Probabilities		Case numbers	
	FE logit		FE Poisson	
	Odds ratio		Multipl. effect	
Inpatient care	Est.	SE	Est.	SE
Overall	0.984***	(0.003)	0.991***	(0.003)
Not PAH	0.989***	(0.003)	0.993*	(0.004)
PAH	0.965***	(0.007)	0.980***	(0.007)
Cardiology	0.957***	(0.012)	0.958***	(0.013)
Pulmonology	0.996	(0.014)	1.021	(0.014)
Diabetes	0.959**	(0.019)	0.949***	(0.020)
Specialist care specific	0.974	(0.018)	0.971	(0.020)
Primary care specific	0.961*	(0.013)	0.983	(0.016)

See Table 1 for details

***$$p<0.01,$$ **$$p<0.05,$$ *$$p<0.1$$

Tables 4 and 5 in "Appendix 4" contain robustness checks. According to Table 4, the slightly negative effect on hospitalization, the more substantial negative effect on PAH and the large positive effect on outpatient care use persist in pooled models or in FE models restricted to those who did not die during the examined period. Table 4 also shows that death was not affected statistically significantly by the new outpatient locations in the medium term.

Table 5 of "Appendix 4" displays that, although the probability of chronic hospitalization (including rehabilitation and nursing services) decreased more than average ($\hbox {OR}=0.95$), the probability of “core” hospitalization (i.e. acute admissions with at least one night in the hospital) also decreased ($\hbox {OR}=0.989$), with marked reduction among acute PAH ($\hbox {OR}=0.95$). According to the table, the results are not governed by the creation of the special 1-day ambulatory services in some treated and control micro-regions, because similar treatment effects are estimated when the sample is restricted to those micro-regions where the newly founded 1-day services had lower than median availability (as measured by per capita case numbers) after 2011.

### Dynamic effects

**Fig. 2:**
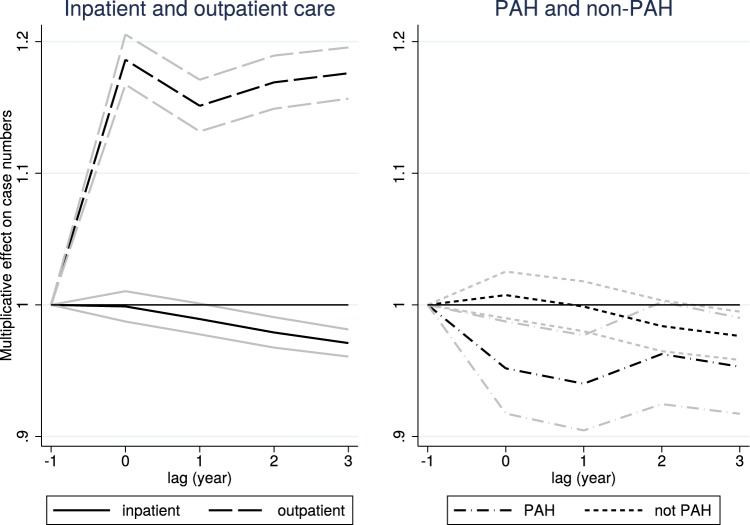
Dynamic effects of the establishment of new outpatient locations on case numbers (with 95% confidence intervals). For exact numbers, see Table 7 of "Appendix 4"

Figure 2 shows the estimated $\beta _{k}$ parameters from the dynamic equation (5). The numerical values of the parameters, along with robustness checks from FE and pooled logit models, are displayed in Table 7 of "Appendix 4". While outpatient case numbers responded quickly to the opening of the new locations, inpatient case numbers reacted with a lag (and decreased by 2–3% after 3 years). According to the right panel of the figure, the lagged reaction was caused by non-PAH case numbers that became statistically significantly reduced by the end of the period, while PAH case numbers decreased right after the opening of the new locations.

The test of the pre-treatment parallel line assumption, detailed in Table 6 of "Appendix 4", shows that inpatient case numbers and its two subcategories changed in a roughly parallel way in the treated and the control micro-regions before the treatment. If anything, hospitalization in the treated micro-regions grew a bit—but statistically not significantly—faster compared to the control micro-regions, so the rate of decrease after the treatment might even be slightly underestimated. Meanwhile, the parallel line assumption is rejected for outpatient case numbers, but the estimated difference in slopes (around 1%) is negligible compared to the change after the treatment (19%).

### Substitution between outpatient and inpatient care

Table 3 shows the estimated structural effects of increased outpatient care use on inpatient care use (as measured by case numbers and expenditures), when the outpatient indicators are instrumented with the treatment dummy. The static estimates suggest that one more (non-laboratory) outpatient case of the patient decreases the number of hospital admissions by about 0.01 and a one HUF increase in outpatient expenditures implies a 0.6 HUF reduction in inpatient expenditures. According to the dynamic models that contain the outpatient indicators and their lags (instrumented by the treatment dummy and its lag), the reduction in the use of inpatient care seems to occur with a lag. This is consistent with the hypothesis that improved outpatient care decreases the need for inpatient care through the better availability of prevention and treatment of chronic diseases.

The above structural results hold only if the instrument is appropriate (relevant and exogenous) in this setting. Relevance is shown by the strength of the first stages, i.e. by the high t values of the treatment dummies in the FE linear models on outpatient care in Table 1 ($t=56.4$ for case numbers and 32.0 for expenditures). The exogeneity of the instrument cannot be proven formally, but, as discussed earlier, the institutional framework (the application procedure of local governments), the lack of other major changes in the health care system during the examined period, and the treatment effect interactions all give indirect evidence for it.

**Table 3:** Structural effects of increased outpatient care indicators on inpatient care indicators

	Parameter		Lagged parameter	
	Est.	SE	Est.	SE
Dependent var.: inpatient case number				
Endogenous explanatory var:				
Outpatient case number	− 0.010***	(0.0034)		
Outpatient case number and its lag	− 0.0058	(0.0035)	− 0.013***	(0.0033)
Dependent var.: inpatient expenditure				
Endogenous explanatory var:				
Outpatient expenditure	− 0.642***	(0.215)		
Outpatient expenditure and its lag	− 0.292	(0.356)	− 0.511*	(0.284)

Cluster-robust standard errors are displayed

Instrumental variables: treatment dummy and its lag. Controls: fixed effects, cubic age, calendar year dummies, and health care supply variables. Model: FE IV.

***$$p<0.01,$$ **$$p<0.05,$$ *$$p<0.1$$

## Conclusions

Our quasi-experimental estimates indicate that bringing outpatient care closer to a previously underserved population may yield considerable effects and not just short-term ones.

As already shown in Elek et al. [5] on a shorter period, indicators of outpatient care use (expenditures and number of visits) increased right after the new outpatient centres were established.

But what is the effect of more access to specialist outpatient services upon inpatient care? Controlling for health care supply variables, fixed effects, and patient age, we find marked substitution effects between outpatient care and hospitalization. As theory predicts, there is smaller effect upon inpatient care when leaving out potentially avoidable hospitalization due to ambulatory care sensitive conditions, but it is larger when concentrating upon potentially avoidable hospitalization. It is especially strong in the two specialisations of diabetes and cardiology. In the case of these two fields, we find corresponding sizeable increases in outpatient laboratory case numbers, strengthening the case that, out of the different theoretical mechanisms, here, the substitution channel of management of chronic health conditions in outpatient care is of great importance. In these specialisations, substitution clearly dominates potential complementation mechanisms.

The dynamics of the effects is also noteworthy: as can be expected the substitution effects are stronger if we allow for a lag of several years for the additional outpatient care to take effect. The substitution effect upon potentially avoidable hospitalization (PAH) is exerted more rapidly than upon hospitalization for the other diagnostic groups, indicating that, in those specialisations, direct substitution mechanisms are present. We interpret the fact that, with a lag of several years, the substitution effect upon non-PAH also becomes significant as a sign that, in addition to prevention due to the early detection, other, slower mechanisms of substitution, notably, better management of chronic conditions are also present. This suggests the presence of medium-term health benefits, although we cannot measure them directly using the available data. Thus, in terms of health policy implications, while we have no conclusive evidence of bringing outpatient care closer upon health outcomes, those effects are likely to be positive.

Finally, the effects concerning expenditures are also significant and sizable. Even though the official Hungarian reimbursement fees may not exactly reflect variable social costs of the treatment (and fix costs are not addressed at all), we consider it remarkable that, according to our estimates, the extra (variable) cost of additional outpatient care (HUF 1300) is partially cancelled out by savings in financing the hospitalization of the patients in question (HUF 800).

What is the external validity of our results vis-a-vis other countries? We can only speculate, but it stands to reason that the more similar the institutional and incentive framework of a healthcare provision to that of Hungary, the more likely that our findings carry over. Thus, we expect that post-communist countries with Semashko-type healthcare setup are the most likely to exhibit a similar substitution relationship between outpatient and inpatient care, but the relationship that we find could also be true for the other countries with single-payer healthcare systems. In as much as our findings for potentially avoidable hospitalization reflect the medical reality of managing chronic health conditions in outpatient vs. inpatient care, they should hold in all the developed countries.

## Appendix 1: Potentially avoidable hospitalization (PAH)

**Table:**

Category	ICD-10 code
Cardiology	
Angina	I20, I240, I248, I249, I250, R072, R073, R074, Z034, Z035
Congestive heart failure	I11, I130, I255, I50, J81
Hypertension	I10, I1191
Pulmonology	
Asthma	J450, J451, J458, J459, J46
COPD	J20, J40, J41, J42, J43, J44, J47
Diabetes	E10, E11, E12, E13, E14
Specialist care specific	
Ear, nose, throat infections	H66.0, H66.1, H66.2, H66.3, H66.4, H66.9, H67, J02, J03, J040, J06, J312
Convulsions, epilepsy	G253, G40, G41, O150, O151, O152, O159, R560, R568
Dental conditions	A690, K02, K03, K04, K05, K06, K08, K098, K099, K12, K13
Pelvic inflammatory disease	N70, N73, N74
Perforated/bleeding ulcer	K20, K210, K219, K221, K226, K250, K251, K252, K254, K255, K256, K260, K261, K262, K264, K265, K266, K270, K271, K272, K274, K275, K276, K280, K281, K282, K284, K285, K286, K920, K921, K922
Pyelonephritis	N10, N11, N12, N136, N159, N300, N308, N309, N390
Primary care specific	
Cellulitis	I891, L01, L02, L03, L04, L080, L088, L089, L88, L980
Gangrene	R02
Dehydration, gastroenteritis	A020, A04, A059, A072, A080, A081, A083, A084, A085, A09, K52
Influenza, pneumonia	A481, A70, J10, J11, J12, J13, J14, J153, J154, J157, J159, J160, J168, J181, J182, J188, J189
Iron or other nutr. def. anaemia	D500, D508, D509, D510, D511, D512, D513, D518, D520, D521, D528, D529, D531, D571, D580, D581, D590, D591, D592, D599, D601, D608, D609, D610, D640, D641, D642, D643, D644, D648
Nutritional deficiency	E40, E41, E42, E43, E550, E643
Other vaccine prev. diseases	A35, A36, A37, A80, B05, B06, B161, B169, B180, B181, B26, G000, M014

Only primary diagnoses were considered, because secondary diagnoses were not available

Age was restricted to at least 2 years

Categorization is based on Purdy et al. [18]

## Appendix 2: Missing inpatient stays in 2015

The original sample refers to only those inpatient events that started and also terminated within 2008–2015, and therefore, some inpatient stays are missing for 2015. All figures in the paper show adjusted data for 2015 by assuming that inpatient events with year of discharge different from year of admission constituted the same share of all inpatient events in 2015 as in 2013–2014. This adjustment increases inpatient case numbers by only 1.2% but inpatient expenditures by 4.5% for 2015, because longer and, hence, more expensive inpatient stays are more likely to carry over to the next year than shorter ones.

The estimation results are essentially unaffected by this sample restriction. If a small share of inpatient events is missing randomly in 2015 in the treated and the control group, the selection effect is completely captured by the calendar year dummy in the FE Poisson model because of its multiplicative structure, and the situation is similar in the FE logit model, because the modelled probabilities are small.

In principle, the FE linear model of the expenditures may be more sensitive to the sample restriction. However, if we impute the additional 4.5% of expenditures for 2015 using the patterns of the previous years, it only changes the elasticity in Table 3 by about 0.01.

## Appendix 3: Health care supply variables

We control for local health care supply with the following variables in our regressions.

First, possible changes in inpatient capacities in the micro-region or the wider region may have influenced hospitalization rates differently in the treated and control groups. Apart from the micro-region of Szikszó (which was excluded from the control group, exactly because acute inpatient care was abolished there, implying a sudden reduction in the rate of hospitalization for its inhabitants), the other examined micro-regions did not have substantial inpatient capacities (hospital beds) throughout the whole observed period, so there is no need to control for inpatient supply on the micro-regional level. At the same time, we control for the logarithm of the wider regional (county-) level number of hospital beds in our regressions.

Second, we control for the availability of GP care using the ratio of unfilled GP practices in the settlement of the individual.

Third, at the time of the establishment of the new outpatient units, special 1-day ambulatory services started to operate in some treated and control micro-regions, which were aimed at providing certain treatments in internal care, neurology, and physiotherapy at the ambulatory level instead of the hospital level. These new services may have had some subtle substitution effect between outpatient and inpatient care. We control for their local availability using the annual micro-regional per capita level of the number of 1-day ambulatory cases in our regressions. Since such services were established only in around half of the treated micro-regions (and also in some control micro-regions), this local availability proxy can be included in our models. We also perform robustness checks (see Table 5 in "Appendix 4") to show that our results are not governed by these 1-day ambulatory services.

## Appendix 4: More details of the estimated models

See Tables 4, 5, 6, 7, 8, 9 and 10.

**Table 4:** Estimates from pooled models and restricted FE models

	Pooled models				Restricted FE models			
	Probabilities		Case numbers		Probabilities		Case numbers	
	Logit		Poisson		Logit		Poisson	
	Odds ratio		Multipl. effect		Odds ratio		Multipl. effect	
	Est.	SE	Est.	SE	Est.	SE	Est.	SE
Death	1.012	(0.017)						
Inpatient care								
Overall	0.994	(0.005)	0.986**	(0.006)	0.985**	(0.006)	0.981***	(0.006)
Not PAH	1.003	(0.005)	0.994	(0.007)	0.998	(0.006)	0.989	(0.007)
PAH	0.939***	(0.012)	0.936***	(0.011)	0.916***	(0.013)	0.936***	(0.014)
Outpatient care								
Overall non-lab.	1.169***	(0.004)	1.181***	(0.004)	1.231***	(0.005)	1.192***	(0.004)
Overall lab.	1.078***	(0.004)	1.124***	(0.006)	1.109***	(0.005)	1.147***	(0.006)

Probability of not PAH refers to the probability that all inpatient stays are not PAH in a year

See Table 1 for baseline probabilities and case numbers. Baseline probability of death: 1.2%

Cluster-robust standard errors are displayed for all models apart from FE logit

Controls for pooled models: cubic age interacted with gender, calendar year dummies, micro-region of residence, and health care supply variables

Controls for FE models: fixed effects, cubic age, calendar year dummies, and health care supply variables

Restricted FE models are estimated on the sample of those who did not die in the examined period

Number of observations: 7,412,000. Number of periods: 8. Number of people: 1,037,000

*PAH* potentially avoidable hospitalization

***$$p<0.01,$$ **$$p<0.05,$$ *$$p<0.1$$

**Table 5:** Robustness checks of the treatment effect on inpatient probabilities

	Odds ratio	
	Est.	SE
Acute and chronic care		
Acute care (excluding 1-day care)	0.989*	(0.0061)
Acute PAH	0.954***	(0.013)
Cardiology	0.949**	(0.023)
Pulmonology	0.992	(0.030)
Diabetes	0.923**	(0.035)
Specialist care specific	1.010	(0.036)
Primary care specific	0.969	(0.026)
Chronic care	0.949***	(0.015)
Sample restricted to micro-regions with low availability of special 1-day services		
Overall hospitalization	0.989	(0.0069)
PAH	0.950***	(0.015)

Controls: see Table 1. Model: FE logit

In the lower panel, the sample was restricted to those micro-regions that had lower than median per capita case number of special 1-day ambulatory services after 2011. It contains the population of 10 treated and all but one control micro-regions

***$$p<0.01,$$ **$$p<0.05,$$ *$$p<0.1$$

**Table 6:** Testing the parallel line assumption on case numbers for years 2008–2010

	Multiplicative interaction terms of treated vs. control group with years				*p* value of both terms $= 1$
	2008 vs. 2010		2009 vs. 2010		
	Est.	SE	Est.	SE	
Overall non-lab outpatient care	0.984***	(0.0044)	0.994	(0.0042)	0.001
Overall inpatient care	0.984*	(0.0091)	0.995	(0.0085)	0.174
PAH	0.972	(0.020)	0.968*	(0.018)	0.188
Not PAH	0.984	(0.0099)	0.999	(0.0093)	0.170

Cluster-robust standard errors are displayed

Controls: see Table 1. Model: FE Poisson

***$$p<0.01,$$ **$$p<0.05,$$ *$$p<0.1$$

**Table 7:** Dynamic effects of the establishment of new outpatient locations

	0 year		1 year		2 years		3 + years	
	Est.	SE	Est.	SE	Est.	SE	Est.	SE
FE Poisson for outpatient case numbers								
Overall non-lab	1.186***	(0.0098)	1.151***	(0.010)	1.169***	(0.010)	1.176***	(0.010)
FE Poisson for inpatient case numbers								
Overall	0.999	(0.0059)	0.989*	(0.0060)	0.979***	(0.0059)	0.971***	(0.0053)
PAH	0.952***	(0.018)	0.940***	(0.018)	0.963*	(0.020)	0.953**	(0.019)
Not PAH	1.007	(0.0090)	0.999	(0.0097)	0.984	(0.0098)	0.976**	(0.0093)
FE logit for inpatient probabilities								
Overall	1.005	(0.0090)	0.993	(0.0091)	0.975***	(0.0089)	0.973***	(0.0079)
PAH	0.928***	(0.019)	0.926***	(0.019)	0.936***	(0.019)	0.929***	(0.017)
Pooled logit for inpatient probabilities								
Overall	1.016**	(0.0075)	0.995	(0.0077)	0.980***	(0.0076)	0.984**	(0.0069)
PAH	0.943***	(0.016)	0.931***	(0.016)	0.940***	(0.017)	0.927***	(0.015)

Controls: see Tables 1, 2, 3 and 4

***$$p<0.01,$$ **$$p<0.05,$$ *$$p<0.1$$

**Table 8:** Descriptive statistics of inpatient dependent variables

	Control micro-regions						Treated micro-regions					
	2008–2009		2010–2012		2013–2015		2008–2009		2010–2012		2013–2015	
	Mean	SD	Mean	SD	Mean	SD	Mean	SD	Mean	SD	Mean	SD
Inpatient probabilities (%)												
Overall	13.55	34.22	13.22	33.87	13.13	33.77	13.64	34.32	13.38	34.05	13.04	33.68
Not PAH	12.07	32.58	11.82	32.28	11.81	32.27	12.15	32.67	12.00	32.49	11.76	32.21
PAH	2.52	15.66	2.40	15.31	2.29	14.96	2.48	15.56	2.37	15.20	2.19	14.65
Cardiology	0.83	9.08	0.79	8.88	0.80	8.89	0.77	8.77	0.74	8.59	0.71	8.38
Pulmonology	0.61	7.80	0.60	7.71	0.54	7.30	0.62	7.84	0.57	7.50	0.53	7.25
Diabetes	0.35	5.90	0.27	5.21	0.22	4.65	0.37	6.03	0.30	5.43	0.23	4.74
Specialist care spec.	0.32	5.64	0.31	5.55	0.31	5.58	0.29	5.35	0.29	5.41	0.29	5.42
Primary care spec.	0.58	7.63	0.60	7.75	0.59	7.68	0.60	7.75	0.64	7.95	0.59	7.67
Inpatient case numbers (per 100 inhabitants)												
Overall	21.68	78.31	21.18	75.98	21.13	77.32	21.58	75.79	21.37	75.84	20.71	74.65
Not PAH	18.61	72.62	18.23	70.17	18.35	72.02	18.60	70.33	18.52	70.41	18.08	69.65
PAH	3.07	22.31	2.95	22.10	2.78	21.20	2.98	21.47	2.85	21.15	2.62	20.32
Cardiology	0.96	11.67	0.92	11.49	0.92	11.55	0.89	11.09	0.86	11.00	0.81	10.79
Pulmonology	0.79	12.17	0.77	12.05	0.69	11.22	0.76	11.22	0.70	10.8	0.64	10.36
Diabetes	0.40	7.50	0.31	6.56	0.24	5.68	0.41	7.35	0.33	6.66	0.25	5.77
Specialist care spec.	0.32	6.23	0.32	6.44	0.32	6.30	0.30	6.11	0.31	6.26	0.30	6.05
Primary care spec.	0.60	8.39	0.63	8.79	0.61	8.53	0.63	8.91	0.66	9.05	0.62	9.03
Inpatient expenditures (1000 HUF/inhabitant)												
Overall	33.14	157.58	32.35	159.07	32.60	158.06	32.65	154.41	32.40	153.5	31.84	155.89
*N*. of obs.	944,550		1,427,989		1,426,575		893,343		1,356,140		1,363,032

**Table 9:** Descriptive statistics of outpatient dependent variables

	Control micro-regions						Treated micro-regions					
	2008-20-09		2010–2012		2013–2015		2008–2009		2010–2012		2013–2015	
	Mean	SD	Mean	SD	Mean	SD	Mean	SD	Mean	SD	Mean	SD
Outpatient probabilities (%)												
Overall non-lab	56.32	49.60	54.83	49.77	53.20	49.90	54.88	49.76	55.50	49.7	55.66	49.68
Cardiology	6.09	23.92	5.75	23.28	5.64	23.07	6.40	24.47	6.45	24.57	6.87	25.29
Pulmonology	4.09	19.79	4.35	20.40	4.18	20.02	4.10	19.83	4.49	20.7	4.91	21.61
Diabetes	2.08	14.28	2.23	14.78	2.44	15.43	2.03	14.11	2.36	15.18	2.68	16.15
Overall lab	30.29	45.95	31.55	46.47	32.05	46.67	30.12	45.88	31.98	46.64	33.57	47.22
Cardiology	1.74	13.07	1.62	12.63	1.66	12.77	1.49	12.13	2.47	15.53	3.08	17.29
Pulmonology	0.33	5.73	0.33	5.76	0.32	5.67	0.32	5.66	0.34	5.83	0.32	5.65
Diabetes	0.82	9.01	0.75	8.64	0.74	8.57	0.54	7.32	0.94	9.64	1.15	10.64
Outpatient case numbers (per 100 inhabitants)												
Overall non-lab	297.38	594.18	291.16	580.61	290.65	589.24	280.47	549.11	303.66	591.37	329.49	645.38
Cardiology	11.98	62.00	11.16	62.06	11.13	66.01	12.76	65.12	13.38	72.94	14.49	73.89
Pulmonology	8.17	59.19	8.95	68.03	8.67	68.13	7.92	54.26	8.57	58.47	9.06	61.45
Diabetes	5.11	46.47	5.19	45.74	5.51	45.96	5.07	45.92	5.69	47.98	6.65	52.93
Overall lab	99.61	329.79	106.96	351.26	107.06	344.69	99.51	346.38	114.18	382.26	124.83	384.04
Cardiology	2.93	32.17	3.00	38.19	2.96	34.56	2.77	33.17	4.24	41.95	5.26	42.62
Pulmonology	0.78	32.41	0.74	22.78	0.73	25.88	0.54	12.5	0.62	15.69	0.56	15.10
Diabetes	1.50	21.87	1.33	19.44	1.31	19.21	1.07	20.04	1.67	22.92	2.17	26.07
Outpatient expenditures (1000 HUF/inhabitant)												
Overall	9.54	25.51	9.76	27.75	10.38	28.19	9.31	25.94	10.23	27.8	11.90	29.87
*N*. of obs.	944,550		1,427,989		1,426,575		893,343		1,356,140		1,363,032

**Table 10:** Descriptive statistics of explanatory variables

	Control micro-regions						Treated micro-regions					
	2008–2009		2010–2012		2013–2015		2008–2009		2010–2012		2013–2015	
	Mean	SD	Mean	SD	Mean	SD	Mean	SD	Mean	SD	Mean	SD
Treatment	0.00	0.00	0.00	0.00	0.00	0.00	0.00	0.00	0.45	0.43	1.00	0.00
Age	40.38	22.07	40.32	22.42	40.29	22.64	39.99	21.96	39.96	22.34	39.93	22.59
Squared age/100	21.17	19.38	21.29	19.62	21.36	19.74	20.82	19.17	20.96	19.44	21.05	19.60
Cubic age/1000	126.82	156.15	128.24	158.82	129.10	160.21	123.92	153.90	125.60	156.86	126.65	158.57
Year (2008)	0.50	0.50	0.00	0.00	0.00	0.00	0.50	0.50	0.00	0.00	0.00	0.00
Year (2009)	0.50	0.50	0.00	0.00	0.00	0.00	0.50	0.50	0.00	0.00	0.00	0.00
Year (2010)	0.00	0.00	0.33	0.47	0.00	0.00	0.00	0.00	0.33	0.47	0.00	0.00
Year (2011)	0.00	0.00	0.33	0.47	0.00	0.00	0.00	0.00	0.33	0.47	0.00	0.00
Year (2012)	0.00	0.00	0.33	0.47	0.00	0.00	0.00	0.00	0.33	0.47	0.00	0.00
Year (2013)	0.00	0.00	0.00	0.00	0.33	0.47	0.00	0.00	0.00	0.00	0.33	0.47
Year (2014)	0.00	0.00	0.00	0.00	0.33	0.47	0.00	0.00	0.00	0.00	0.33	0.47
Year (2015)	0.00	0.00	0.00	0.00	0.33	0.47	0.00	0.00	0.00	0.00	0.33	0.47
Special 1-day services	0.00	0.00	0.04	1.49	0.59	1.29	0.00	0.00	1.33	2.84	3.35	3.58
Log hospital beds in county	7.98	0.38	7.98	0.37	7.94	0.34	8.03	0.35	8.02	0.35	7.98	0.33
Unfilled GP practices (%)	6.16	22.02	6.78	22.44	8.6	24.31	2.47	11.98	4.45	17.79	6.32	21.1
*N*. of obs.	944,550		1,427,989		1,426,575		893,343		1,356,140		1,363,032	

Special 1-day services: average case number in the micro-region per 100 inhabitants

Unfilled GP practices: percent in the settlement

## Footnotes

1 The decision to apply for EU-financed expansions had to do with the obligation of the local government to maintain the new outpatient units, financed with fee-for-service point reimbursement, which is a function of the expected number of patients. The latter could be estimated from geography, population density, and the location of other healthcare providers.

2 The previous study of Elek et al. [5], which focused on short-term patterns of outpatient care, used 21 control micro-regions. In the current analysis, we exclude the micro-region of Szikszó, because acute inpatient care was abolished in its hospital during our examined period. The matched inpatient–outpatient data, used in the current study, had not yet been available at the time of writing of the past paper.

3 The data set covers only those people who appeared at least once in outpatient or inpatient care between 2008 and 2015. This is a negligible restriction for two reasons. First, other administrative data (the linked labor-health panel data set processed by the Institute of Economics, Centre for Economic and Regional Studies of the Hungarian Academy of Sciences, see, e.g. Bíró and Elek [2] for its health variables) suggest that less than 2.5% of the (18–74 years old) inhabitants of the examined micro-regions did not appear at all in either outpatient or inpatient care during another 8 year long period (2003–2011). Second, we use fixed-effects Poisson and logit models in our main analysis, and the always zero observations drop out in the estimation of these models.

4 As a robustness check, we also defined PAH following the European Collaboration for Healthcare Optimization (ECHO) project [22], which examines hospitalization due to angina, congestive heart failure, and strictly defined diabetes complications for people at least 40 years old, asthma and COPD for people at least 18 years old, and dehydration complications for people at least 65 years old. Our results do not change substantially when this alternative definition is used.

5 We note that the original data refer to only those inpatient events that started and also terminated within 2008–2015, and therefore, some inpatient stays are missing for 2015. All figures in the paper show the adjusted data for 2015 by assuming that inpatient events with year of discharge different from year of admission constituted the same share of all inpatient events in 2015 as in 2013–2014. This adjustment increases inpatient case numbers by only 1.2%, and does not affect substantially our later results. For details, see "Appendix 2".

6 Individual-level expenditures are approximated based on the financing rules of the Hungarian health care system, but they cannot be calculated precisely from the data at hand, because some financial variables of minor importance are missing. Hence, our results on expenditures only give rough estimates on the financial interactions between outpatient and inpatient care.

7 The nearest substantial hospital is defined as the most frequent place of inpatient stay of the population of a micro-region.

8 This is in line with the short-term results by Elek et al. [5], who could only examine the outpatient data up until 2012 (and inpatient data were not available then) and could not distinguish the various outpatient diagnoses.

9 Comparison of outpatient and inpatient spending is further complicated by the fact that drugs prescribed in ambulatory care are partially financed by out-of-pocket co-payment by the patient, whereas, in inpatient care, the full cost of medication is borne by the hospital and, thus, factored into the amount of the reimbursement. However, aggregate pharmaceutical consumption trends are roughly parallel in the treated and control group, so this effect is negligible.

10 These specifications do not contain the treatment dummy, only the travel time to the nearest location, because the former is not statistically significant when the latter is included in the regression.
